# Leptin Inhibits Glucose Intestinal Absorption via PKC, p38MAPK, PI3K and MEK/ERK

**DOI:** 10.1371/journal.pone.0083360

**Published:** 2013-12-10

**Authors:** Ola El-Zein, Sawsan Ibrahim Kreydiyyeh

**Affiliations:** Department of Biology, American University of Beirut, Beirut, Lebanon; Chang Gung University, Taiwan

## Abstract

The role of leptin in controlling food intake and body weight is well recognized, but whether this is achieved by modulating nutrient absorption is still a controversial issue. The aim of this work was to investigate the direct effect of luminal leptin on glucose intestinal absorption and elucidate for the first time its signaling pathway. Fully differentiated Caco-2 cells grown on transwell filters were used for glucose transport studies. Leptin caused a significant reduction in glucose absorption. Individual and simultaneous inhibition of ERK, p38MAPK, PI3K or PKC abrogated completely the inhibitory effect of leptin. Activating PKC, lead to a stimulatory effect that appeared only when ERK, p38MAPK, or PI3K was inactive. Moreover, leptin increased the phosphorylation of ERK, Akt and p38MAPK. This increase changed into a decrease when p38MAPK and PKC were inactivated individually. Inhibiting ERK maintained the leptin-induced up-regulation of p-Akt and p-p38MAPK while inhibiting PI3K reduced the level of p-ERK and p-Akt but maintained the increase in p-p38MAPK. These results suggest that leptin reduces glucose absorption by activating PKC. Although the latter modulates glucose absorption via a stimulatory and an inhibitory pathway, only the latter is involved in leptin’s action. Active PKC leads to a sequential activation of p38MAPK, PI3K and ERK which exerts an inhibitory effect on glucose absorption. The results reveal a modulatory role of leptin in nutrient absorption in addition to its known satiety inducing effect.

## Introduction

Obesity increases the occurrence of several diseases and is a leading contributor to morbidity and mortality both in developed and developing countries. It is mainly caused by excessive food and energy intake [1].

Since its discovery in 1994, leptin has been recognized as a hormone playing an important role in energy homeostasis [2]. Studies on massively obese mice and human subjects demonstrated that normal production and action of leptin are critical for controlling body weight and adiposity [3-5]. Initially, leptin was known to be secreted by white adipose tissue, and circulates at levels directly proportional to the total amount of fat in the body [6]. However, it is now considered a multifunctional hormone that is produced by various tissues and organs including the placenta [7], kidney [8], salivary glands [9], and stomach [10]. Leptin secreted from the chief cells of the stomach is released into the gastric juice and remains active despite the severe acidic environment [10-13].

Similar to other hormones, leptin exerts its effects by interacting with its receptors (OB-Rs) which are expressed in several peripheral tissues [14-16] including the apical membrane of intestinal epithelial cells [17-19]. 

 The secretion of leptin by the stomach and its entry with chyme into the small intestine suggest that it may play a role in food absorption. Indeed, several studies have shown that leptin interferes with the absorption of some nutrients. It enhances butyrate uptake [18], intestinal transport of fructose [20] and oligopeptides [21], and decreases galactose uptake [22,23] and glutamine transport [24]. Glucose is the major end-product of carbohydrate digestion and a main source of energy for the body. Whether modulation of glucose absorption may be one aspect of the recognized role of leptin in inducing satiety is a question that has not been addressed properly till now.

Literature on this topic is scarce [25] and the few studies undertaken so far focused on the effect of leptin on glucose transport in intestinal loops, or on the change in current in Ussing chambers [26,27]. These studies were conducted thus on tissues containing other cells in addition to the absorptive enterocytes. Consequently, one cannot rule out the possibility of the hormone acting on non-intestinal cells and inducing them to produce factors that interfere with the absorption of glucose by enterocytes. This is why we opted in this work to use Caco-2 cells that provide a model highly used in transport studies. These cells are cancerous colon cells but differentiate when confluent into enterocytes and acquire all their properties. They express in addition leptin receptors [19,21,28,29], and can be cultured on transwell supports giving access to both apical and basolateral membranes [21,29,30]. Their use will allow for the determination of the direct effect of leptin on intestinal cells as well as its mode of action. 

 The aim of this study was to investigate the direct effect of luminal leptin on glucose absorption by fully differentiated Caco-2 cells and to elucidate its signaling pathway, which although highly studied in the central nervous system, is still ill-defined in the peripheral tissues. 

The results might help in the design of new drugs that imitate leptin’s action and contribute to the treatment of obesity and other related disorders.

## Materials and Methods

### Materials

 Human leptin was purchased from Biovision, CA, USA. Rabbit anti-ERK1/2 polyclonal antibody was purchased from Promega, WI, USA. Rabbit anti-p-p44/42 MAPK (ERK1/2) monoclonal antibody was purchased from Cell Signaling, MA, USA. Rabbit anti-Akt1/2/3, anti-p- Akt1/2/3, anti-p38α, anti-p-p38α polyclonal antibodies, and anti-rabbit IgG horse raddish peroxidase (HRP) conjugated were purchased from Santa Cruz, CA, USA. Protease inhibitors cocktail tablets were purchased from Boehringer Mannheim, Germany. Enhanced Chemiluminescence (ECL) kit was obtained from Santa Cruz, CA, USA. Nitrocellulose membranes, Biorad protein assay reagent and rainbow marker were purchased from Biorad, California, USA. Dulbecco’s Minimal Essential Medium (DMEM) with 4500 mg L^-1^ Glucose and pyridoxine HCL, Fetal Bovine Serum (FBS), Penicillin/Streptomycin (PS), Trypsin-EDTA, 10x Phosphate Buffered Saline (PBS) without calcium and magnesium were purchased from Sigma, Chemical CO, St. Louis, Missouri, USA. Phorbol-12-myristate-13-acetate (PMA), an activator of PKC, was purchased from CALBIOCHEM, San Diego, California, USA. Wortmannin, PD98059, SB202190, and Calphostin C, respective inhibitors of PI3K, MEK/ERK, p38MAPK and PKC, were also purchased from CALBIOCHEM, San Diego, California, USA. Transwell permeable supports (0.4 µm-pore-sizes, 24 mm-diameter, polyester) were purchased from Corning, MA, U.S.A. Scintiverse BD Cocktail was purchased from Fisher Scientific, Fairlawn, NJ, USA 3-O-methyl-D-glucose ([^14^C] 3OMG) and Mannitol, D-[1-^3^H (N)] were bought from Amersham International Ltd. Amersham, UK. The human colon carcinoma cell line (Caco-2) from a Caucasian male was purchased from American Type Culture Collection (ATCC), VA, USA.

## Methods

### Cell Culture and Treatments

#### a: Culture of Caco-2 Cells

 Caco-2 cells were used at passages 25-35. They were grown in DMEM containing 4500 mg L^-1^ Glucose, sodium pyruvate, 1% Penicillin (100 µg mL^-1^), streptomycin (100 µg mL^-1^), 10% FBS, in a humidified incubator (95% O_2_, 5% CO_2_) at 37°C. The cells were grown either on 100 mm culture plates or on polyester transwell permeable supports (0.4 µm-pore-sizes, 24 mm-diameters) at a density of 120,000 cells mL^-1^ and treated always on the twenty third day after confluence following an overnight starvation. 

#### b: Effect of leptin on glucose absorption by Caco-2 cells

 Leptin (10 nM), 3-O-^14^C methyl D glucose ([^14^C] 3OMG) (0.3 μM, 2.087 GBq mmol^-1^) and D-mannitol-[1-^3^H (N)] (0.44 nM, 455.1 GBq mmol^-1^) were added to the upper well of the transwell inserts on which Caco-2 cells were cultured. Mannitol was added to check for the integrity of the Caco-2 cells monolayer and to confirm junctional tightness. Samples were collected from the lower chamber immediately upon addition of the radioactive tracers and every 10 min thereafter, over a period of 50 min, and were assayed for radioactivity. 

#### c: Determination of the signaling mediators of leptin

The involvement of MEK/ERK, p38MAPK, PI3K, and PKC in the pathway mediating the effect of mucosal leptin on glucose absorption was studied. Starved fully differentiated CaCo-2 cells grown on transwell inserts for 23 days after confluence were pre-treated with a specific inhibitor of each of the above mediators and which are respectively: PD98059 (50 µM dissolved in DMSO), SB202190 (50 µM dissolved in DMSO), wortmannin (100 nM dissolved in DMSO) and Calphostin C (50 nM dissolved in DMSO). All inhibitors were added 15 min before leptin, except wortmannin and Calphostin C which were added respectively 20 min and 60 min prior to leptin. The vehicles were added to the control plates in the same amount and for the same duration. 

 In another set of experiments, the cells were also treated at the same dose and times described above but the four mediators were inhibited simultaneously in different combinations (Calphostin C and SB202190, Calphostin C and wortmannin, PD98059 and wortmannin, PD98059 and SB202190 and wortmannin). In addition, PMA (100 nM dissolved in DMSO), an activator of PKC was also administered either alone or in the individual presence of inhibitors of MEK/ERK (PD98059), or p38MAPK (SB202190) or PI3K (wortmannin). 

### Western Blot Analysis

 On day 23 after confluence, starved fully differentiated CaCo-2 cells grown on 100 mm culture plates were pre-treated, before leptin (10nM, 50 min), with a specific inhibitor of each of the above mediators and which are: PD98059, SB202190, wortmannin and Calphostin C. The treatments were done at the same doses and times as described in part c above. At the end of the incubation period, the plates were washed twice with PBS buffer (pH 7.4) and the cells lysed and homogenized in a polytron (20,000-22,000 rpm) at 4°C after addition of a cocktail of phosphatase inhibitors. Proteins were quantified using the Bio-Rad assay. Equal amounts of proteins were loaded, resolved on 8% (Akt) or 10% (p38MAPK, ERK) SDS polyacrylamide gel and transferred to a nitrocellulose membrane which was then blocked and incubated with a primary p38α, phospho-p38α, Akt1/2/3, phospho-Akt1/2/3, ERK1/2, or phospho-p44/42 MAPK (ERK1/2) antibody followed by an incubation with a goat anti-rabbit secondary horseradish peroxidase (HRP) conjugated IgG. The signal was detected by enhanced chemiluminescence using luminol. Equal loading was checked by GAPDH expression. 

### Statistical Analysis

 Results are reported as means ± SEM and tested for statistical significance by a one-way Analysis of Variance (ANOVA) followed by Tukey-Kramer multiple comparisons test using Instat and Excel Analysis softwares. 

## Results

### Effect of mucosal leptin on glucose absorption by Caco-2 Cells

**Figure 1:**
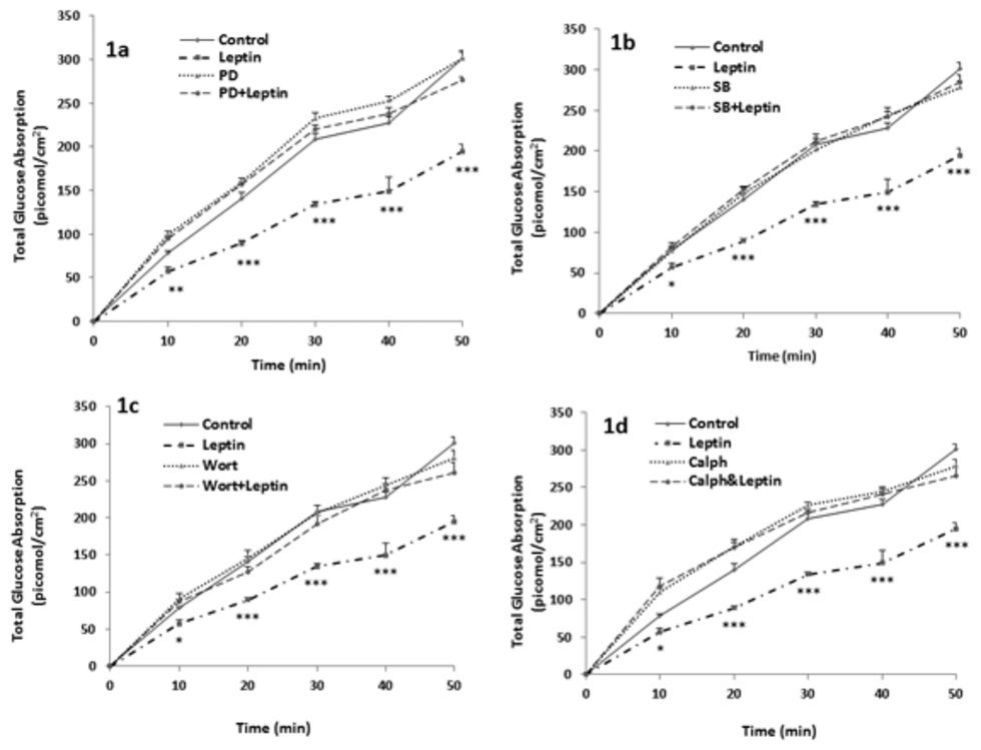
Involvement of MEK/ERK, p38MAPK, PI3K, and PKC in leptin’s action. Effect of leptin (10 nM) on glucose absorption by Caco-2 cells in presence and absence of (a) PD98059, inhibitor of MEK/ERK, (b) SB202190, an inhibitor of p38MAPK, (c) Wortmannin, an inhibitor of PI3K, (d) Calphostin C, an inhibitor of PKC. Absorption of glucose was measured at 10-min intervals over a period of 50 min. Values are means ± SEM of 5 observations. * P<0.05, **P<0.01, ***P<0.001: significantly different from all other treatments.

Mucosal leptin (10 nM) caused a significant reduction in glucose absorption by fully differentiated CaCo-2 cells (23 days after confluence). This decrease appeared at 10 min and was maintained up to 50 min (Figure 1a). 

 The apparent permeability coefficient of mannitol ranged between 6.6 x 10^-10^ cm s^-1^ and 9.5 x 10^-8^ cm s^-1^. Monolayers in which the coefficient was higher than 5 x 10^-7^ cm s^-1^ were considered leaky and were discarded [31]. 

### Involvement of MEK/ERK, p38MAPK, PI3K, and PKC in mucosal leptin action

 When Caco-2 cells were pretreated with PD98059, SB202190, wortmannin, or calphostin C, respective inhibitors of MEK/ERK, p38MAP kinase, PI3-kinase and PKC, the inhibitory effect of leptin on glucose absorption disappeared completely and went back to control values (Figures 1a-1d). 

**Figure 2:**
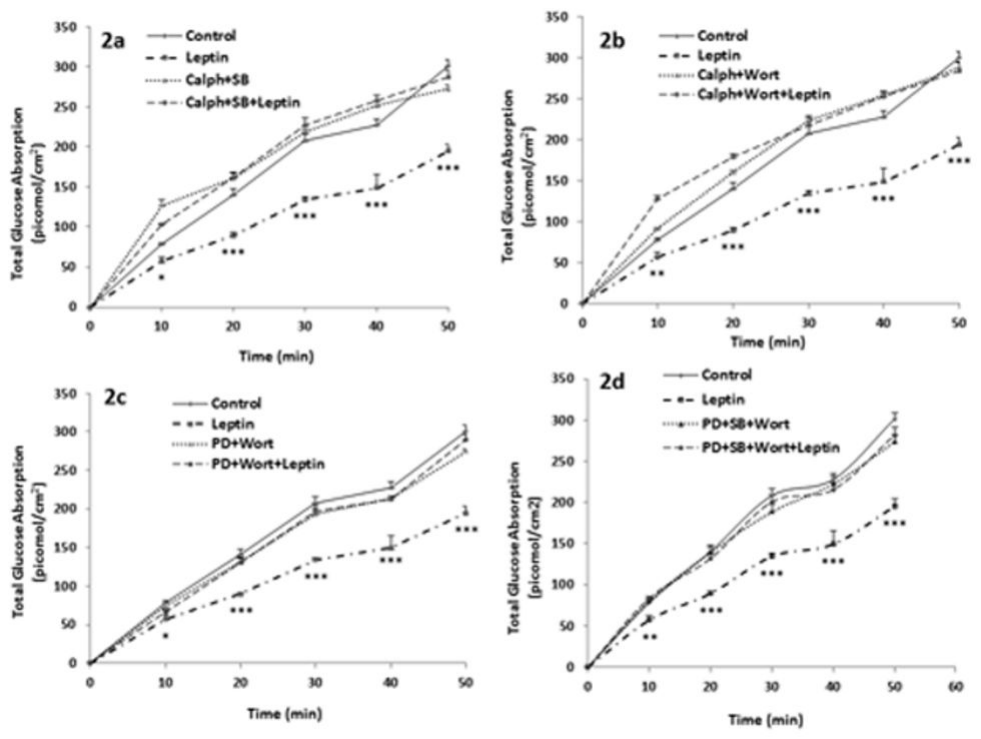
MEK/ERK, p38MAPK, PI3K and PKC act along the same pathway. Effect of leptin (10 nM) on glucose absorption by Caco-2 cells in presence and absence of (a) Calphostin C + SB202190, inhibitors of PKC and p38MAPK respectively, (b) Calphostin C + Wortmannin, respective inhibitors of PKC and PI3K, (c) PD98059 + Wortmannin, inhibitors of MEK/ERK and PI3K respectively, (d) PD98059 + SB202190 + Wortmannin, respective inhibitors of MEK/ERK, p38MAPK and PI3K. Absorption of glucose was measured at 10-min intervals over a period of 50 min. Values are means ± SEM of 5 observations. * P<0.05, **P<0.01, ***P<0.001: significantly different from all other treatments.

 The effect of leptin on glucose absorption was similarly abrogated when Caco-2 cells were pre-treated simultaneously with calphostin C and SB202190, or calphostin C and wortmannin. Each combination of inhibitors alone did not have any effect on glucose absorption (Figures 2a & 2b). 

Leptin could not exert its effect on glucose absorption in the simultaneous inhibition of MEK/ERK and PI3K, or the simultaneous inhibition of MEK/ERK, p38MAPK and PI3K (Figures 2c & 2d).

**Figure 3:**
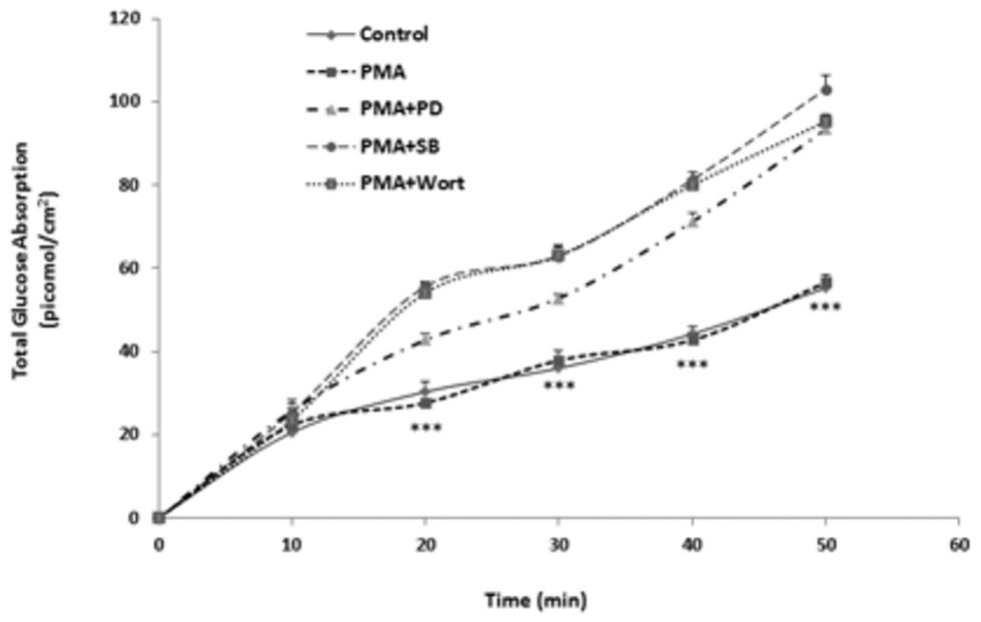
Effect of PKC activation with PMA in the individual presence of inhibitors of MEK/ERK (PD98059), or p38MAPK (SB202190) or PI3K (Wortmannin). Absorption of glucose was measured at 10-min intervals over a period of 50 min. Values are means ± SEM of 5 observations. ***P<0.001: significant difference from PMA + PD98059 as well as from PMA + SB202190 and PMA + Wortmannin.

PMA, an activator of PKC, did not have any effect on glucose absorption (Figure 3). However, the activation of PKC with a simultaneous inhibition of each of MEK/ERK, p38MAPK or PI3K resulted in a significant increase in glucose absorption (Figure 3). 

### Effect of mucosal leptin on the level of p-ERK1/2, p-Akt1/2/3 and p-p38MAPKα

Leptin increased the phosphorylation of ERK (p-ERK), Akt (p-Akt) and p38MAPK (p-p38MAPK) (Figures 4.1-4.3).

**Figure 4:**
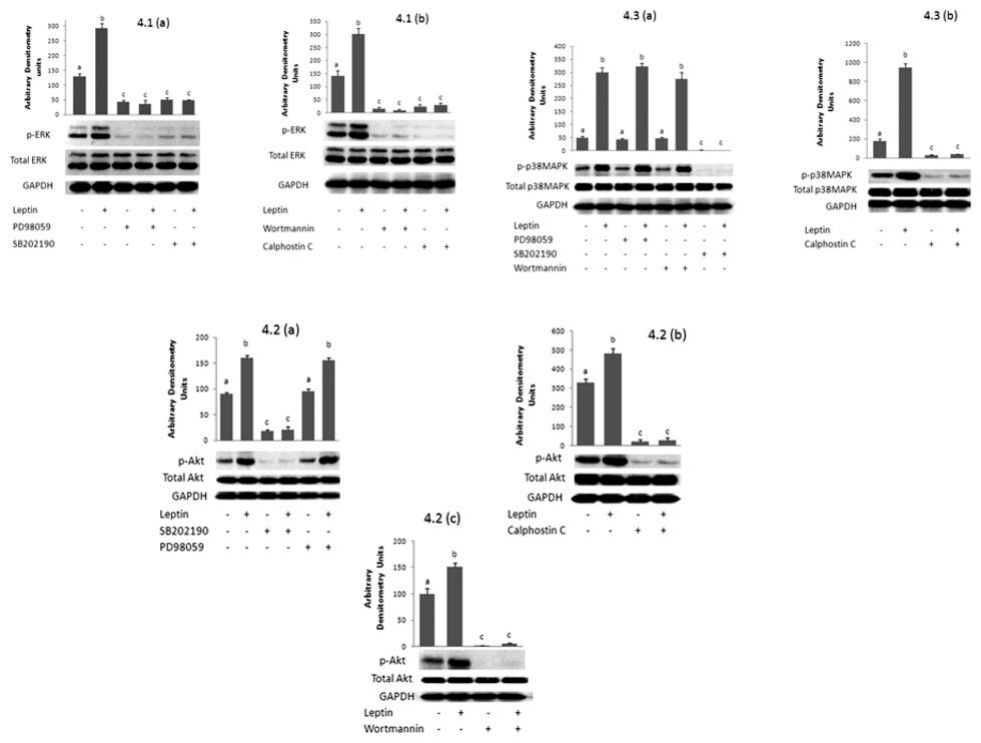
Positioning ERK, PI3K, p38MAPK, and PKC, with respect to each other. Effect of leptin (10 nM, 50 min) on 4.1 phosphorylated ERK when (a) ERK and p38MAPK are inhibited with PD98059 and SB202190 respectively, (b) PI3K and PKC are inhibited respectively with Wortmannin and Calphostin C. 4.2 phosphorylated Akt when (a) ERK and p38MAPK are inhibited with PD98059 and SB202190 respectively, (b) PKC is inhibited with Calphostin C (c) PI3K is inhibited with Wortmannin, 4.3 phosphorylated p38MAPK when (a) ERK, p38MAPK and PI3K are inhibited respectively with PD98059, SB202190 and Wortmannin, (b) PKC is inhibited with Calphostin C. All the results are representative of an experiment repeated 3 times. Bands were quantified using the gel- Pro Analyzer 3.0 software and are accompanied by their densitometric analysis. The intensity of the bands is reported as “arbitrary densitometry units”. Letters on top of the bars indicate significant difference. Bars having different letters are significantly different from each other (P<0.01).

This increase changed into a decrease in presence of SB202190, an inhibitor of p38MAPK (Figures 4.1(a), 4.2(a), & 4.3(a)). Leptin did not have, however, any effect on the level of total ERK, Akt or p38MAPK (Figures 4.1-4.3).

The leptin-induced increase in the phosphorylated forms of Akt and p38MAPK still appeared when MEK/ERK was inhibited (Figures 4.2(a) & 4.3(a)). No p-ERK could be detected in presence of PD98059 confirming its specific inhibitory effect on the kinase (Figure 4.1(a)).

When PI3K was inhibited with wortmannin, leptin caused instead of an increase, a decrease in the level of p-ERK (Figure 4.1(b)) and p-Akt (Figure 4.2(c)), but still enhanced the phosphorylation of p38MAPK (Figure 4.3(a)). Inhibiting PKC also changed the effect of leptin into a decrease in the level of p-ERK, p-Akt and p-p38MAPK (Figures 4.1(b), 4.2(b) & 4.3(b) respectively).

## Discussion

This work demonstrated a significant inhibitory effect of mucosal leptin on glucose absorption in fully differentiated Caco-2 cells. Because extracellular signal-regulated kinase (ERK), p38 mitogen-activated protein kinase (p38MAPK), phosphatidylinositol-3-OH kinase (PI3K) and protein kinase C (PKC) are known to be involved in the regulation of glucose transport [32-36] , we hypothesized that these four mediators might be involved in the signaling pathway underlying the inhibitory effect of the hormone. This hypothesis was confirmed when inhibiting individually ERK, p38MAPK, PI3K or PKC abrogated completely the effect of leptin (Figures 1a-1d). Such an involvement has been observed by other workers in other cells. Van den Brink et al. [37], reported a leptin induced increase in p38MAPK phosphorylation in human mononuclear cells. Leptin was found also to stimulate PI3K in different tissues and organs such as HepG2 cells [38], pancreatic β cells [39], fibroblasts [40], macrophages [41], C2C12 muscle cells [42] and insulinoma cells [43]. Similarly, ERK1 kinase activity and endogenous ERK2 phosphorylation were increased by leptin in COS cells, hamster ovary cell lines and human embryonic kidney cells [44,45]. The effect on PKC was however cell-dependent. While activation of PKC was noted in kidney epithelial cells [46], inhibition was observed in pancreatic islets [47]. 

 The results imply also that all four kinases involved in leptin’s action are along the same pathway, or else, a partial effect would have remained when every mediator was inhibited individually. To confirm that the four mediators act along the same pathway, they were inhibited simultaneously in different combinations. Leptin could not exert its effect when PKC was inhibited simultaneously with p38MAPK or simultaneously with PI3K (Figures 2a & 2b). Thus, although in the first combination MEK/ERK and PI3K were still active, leptin could not act. Similarly, having only MEK/ERK and p38MAPK active was not enough to see leptin’s effect. Likewise, inhibiting MEK/ERK and PI3K simultaneously leaving PKC and p38MAPK active or inhibiting simultaneously MEK/ERK, p38MAPK and PI3K leaving PKC active abolished the effect of leptin (Figures 2c & 2d). The findings indicate that all four mediators need to be active and confirm our previous conclusion that all of them are along the same pathway. 

As the above results indicate that leptin acts via activation of PKC, we attempted to imitate the effect of leptin by activating PKC with PMA. To our surprise, PMA did not have any effect on glucose absorption, but caused a significant increase when administered simultaneously with PD98059, an inhibitor of ERK (Figure 3). These findings stipulate that an active ERK inhibits glucose absorption and by so doing masks and counteracts a stimulatory effect of PKC that cannot appear except when ERK is inactive. Thus, PKC seems to act on glucose absorption via two pathways: an inhibitory pathway mediated via ERK and a stimulatory pathway mediated via an unknown effector (X) activated by PKC and which increases glucose absorption. Leptin may be activating PKC and inhibiting the mediator X, cancelling thus the stimulatory pathway and leaving only the inhibitory pathway involving p38MAPK, PI3K and ERK active. The same stimulatory effect was observed when Caco-2 cells were pretreated simultaneously with both SB202190 (inhibitor of p38MAPK) and PMA (Figure 3), or with wortmannin (inhibitor of PI3K) and PMA (Figure 3) confirming the presence of all four kinases along the same pathway that leads to an inhibition of glucose absorption. 

Since all four mediators are along the same pathway, the positioning of one mediator with respect to the other needs to be determined. The activation of each mediator was checked by western blot analysis by determining changes in the level of their phosphorylated active forms or in the phosphorylation of their substrates. Leptin increased the phosphorylation of ERK (Figures 4.1(a) & 4.1(b)) thus activating ERK and confirming the above conclusion. This increase disappeared in presence of PD98059 (Figure 4.1(a)) confirming that PD98059 is a specific pharmacological inhibitor of ERK. Similarly, the leptin induced increase in p-ERK was reduced when p38MAPK was inhibited by SB202190 (Figure 4.1(a)) indicating that p38MAPK is upstream of ERK and acts by activating it. Moreover, p-ERK was also decreased when PI3K or PKC were inhibited individually (Figure 4.1(b)) inferring that both kinases are up-stream of ERK. It can be concluded that ERK is downstream of all other mediators. Our results are in line with those of Berti and Gammeltoft [48], who reported in C2C12 muscle cells a leptin-induced increase in glucose uptake that was mediated via activation of ERK2 in a PI3K dependent manner. 

To position PI3K, the effect of leptin on the level of the phosphorylated form of its downstream target, the serine/threonine kinase Akt/PKB, was studied in presence of inhibitors of the different mediators. Akt is activated by phosphorylation on two residues, Thr308 and Ser473. An antibody directed against these residues was used. The level of p-Akt was increased by leptin (Figures 4.2(a), 4.2(b) & 4.2(c) ) which is in accordance with the previous results and conclusions, but remained unaltered when cells were pre-treated with an inhibitor of MEK/ERK, indicating that ERK is downstream of PI3K (Figure 4.2(a)). However the leptin induced increase in Akt phosphorylation was reduced when p38MAPK and PKC were individually inhibited (Figures 4.2(a) & 4.2(b)) inferring that both are situated up-stream of PI3K and exert a stimulatory effect on PI3K. In presence of its pharmacological inhibitor, wortmannin, the level of p-Akt was decreased (Figure 4.2(c)), confirming thus that wortmannin is a specific inhibitor of PI3K. 

Since ERK was found to be the most downstream effector and to be directly activated by PI3K which is in turn activated by both PKC and p38MAPK, the positioning of p38MAPK and PKC with respect to each other still needs to be investigated. Leptin increased the phosphorylation of p38MAPK (Figures 4.3(a) & 4.3(b)). This increase was not affected by inhibition of MEK/ERK or PI3K (Figure 4.3(a)), indicating again that p38MAPK is upstream of both PI3K and ERK. However, p-p38MAPK was decreased in presence of SB202190 (Figure 4.3(a)), confirming that SB202190 is a direct pharmacological inhibitor of p38MAPK. Inhibiting PKC reduced the level of p-p38MAPK (Figure 4.3(b)) indicating that PKC acts upstream of p38MAPK and activates it. 

**Figure 5:**
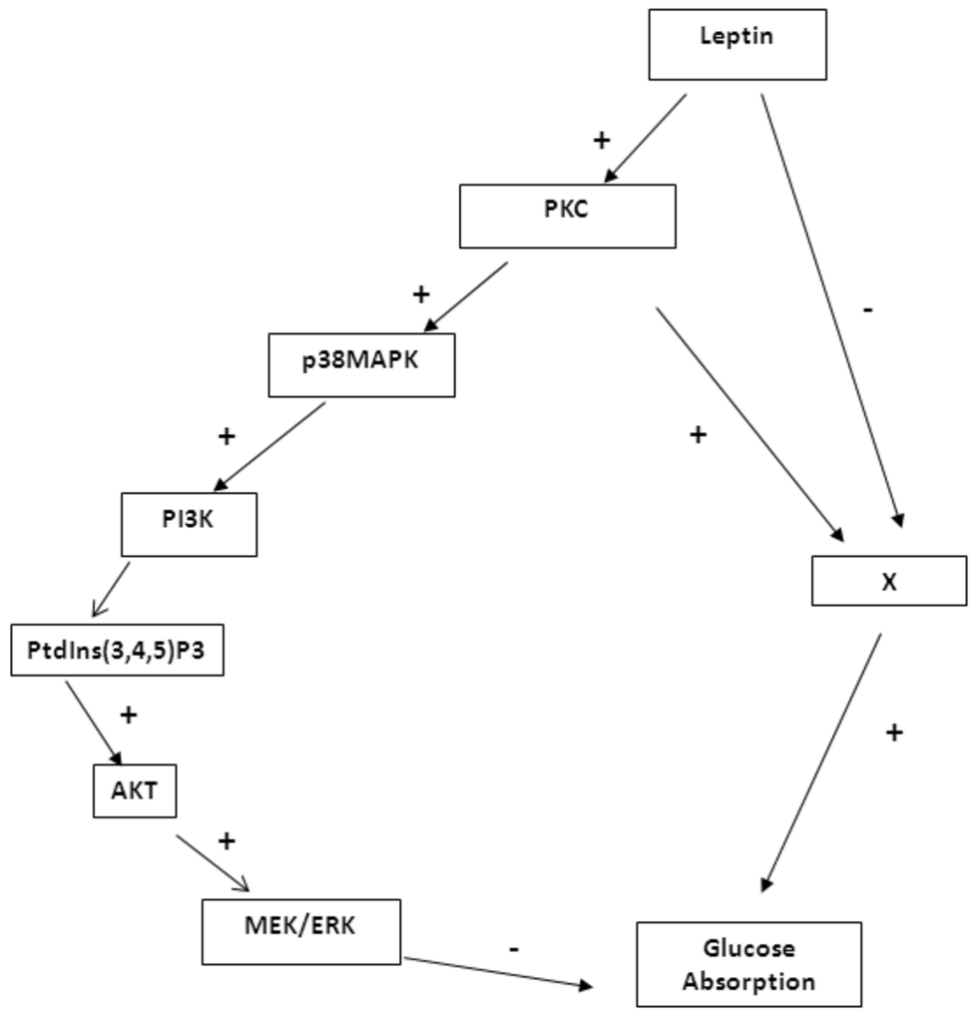
Proposed signaling pathway for leptin action. (-): inhibitory effect; (+): stimulatory effect.

It can be concluded that leptin inhibits glucose absorption by activating PKC which acts via a stimulatory and inhibitory pathway. Leptin inhibits at the same time the stimulatory pathway and leaves only the inhibitory one active resulting thus in a reduction in glucose absorption. This inhibitory pathway involves activation of PKC which activates p38MAPK. The latter activates PI3K which eventually activates ERK. An active ERK exerts an inhibitory effect on glucose absorption. Figure 5 shows the suggested pathway. 

This work determined for the first time the direct effect and signaling pathway of mucosal leptin on glucose absorption by differentiated Caco-2 cells grown on transwell inserts. The identity of the mediator X needs still to be determined in a future work. These results suggest that leptin may induce weight loss not only by reducing appetite, but also by interfering with nutrient absorption. 

The findings provide experimental evidence supporting the benefit of leptin as a complementary therapeutic agent in the treatment of obesity and other related disorders. 

## References

[1] Barness LA, Opitz JM, Gilbert-Barness E (2007) Obesity: genetic, molecular, and environmental aspects. Am J Med Genet A 143A: 3016–3034. doi:10.1002/ajmg.a.32035. PubMed: 18000969. 18000969

[2] Zhang Y, Proenca R, Maffei M, Barone M, Leopold L et al. (1994) Positional cloning of the mouse obese gene and its human homologue. Nature 372: 425- 432. doi:10.1038/372425a0. PubMed: 7984236. 7984236

[3] Halaas JL, Gajiwala KS, Maffei M, Cohen SL, Chait BT et al. (1995) Weight-reducing effects of the plasma protein encoded by the obese gene. Science 269: 543–546. doi:10.1126/science.7624777. PubMed: 7624777. 7624777

[4] GreGreen ED, Maffei M, Braden VV, Proenca R, DeSilva U et al. (1995) The human obese (OB) gene: RNA expression pattern and mapping on the physical, cytogenetic, and genetic maps of chromosome 7. Genome Res 5: 5–12. doi:10.1101/gr.5.1.5. PubMed: 8717050. 8717050

[5] Sinha MK, Opentanova I, Ohannesian JP, Kolaczynski JW, Heiman ML et al. (1996) Evidence of free and bound leptin in human circulation. Studies in lean and obese subjects and during short-term fasting. J Clin Invest 98: 1277–1282. doi:10.1172/JCI118913. PubMed: 8823291. 8823291 PMC507552

[6] Considine RV, Sinha MK, Heiman ML, Kriauciunas A, Stephens TW et al. (1996) Serum immunoreactive-leptin concentrations in normal-weight and obese humans. N Engl J Med 334: 292–295. doi:10.1056/NEJM199602013340503. PubMed: 8532024. 8532024

[7] Masuzaki H, Ogawa Y, Sagawa N, Hosoda K, Matsumoto T et al. (1997) Nonadipose tissue production of leptin: leptin as a novel placentaderived hormone in humans. Nat Med 3: 1029–1033. doi:10.1038/nm0997-1029. PubMed: 9288733. 9288733

[8] Martínez-Ansó E, Lostao MP, Martinez JA (1999) Immunohistochemical localization of leptin in rat kidney. Kidney Int 55: 1129–1130. PubMed: 10215465. 10215465 10.1046/j.1523-1755.1999.0550031128.x

[9] De Matteis R, Puxeddu R, Riva A, Cinti S ( 2002) Intralobular ducts of human major salivary glands contain leptin and its receptor. J Anat 201: 363–370. doi:10.1046/j.0021-8782.2002.00106.x. PubMed: 12448771. 12448771 PMC1570946

[10] Sobhani I, Bado A, Vissuzaine C, Buyse M, Kermorgant S et al. (2000) Leptin secretion and leptin receptor in the human stomach. Gut 47: 178–183. doi:10.1136/gut.47.2.178. PubMed: 10896907. 10896907 PMC1727985

[11] Bado A, Levasseur S, Attoub S, Kermorgant S, Laigneau JP et al. (1998) The stomach is a source of leptin. Nature 394: 790–793. doi:10.1038/29547. PubMed: 9723619. 9723619

[12] Cammisotto PG, Renaud C, Gingras D, Delvin E, Levy E et al. (2005) Endocrine and exocrine secretion of leptin by the gastric mucosa. J Histochem Cytochem 53: 851–860. doi:10.1369/jhc.5A6620.2005. PubMed: 15995144. 15995144

[13] Guilmeau S, Buyse M, Tsocas A, Laigneau JP, Bado A (2003) Duodenal leptin stimulates cholecystokinin secretion: evidence of a positive leptin-cholecystokinin feedback loop. Diabetes 52: 1664–1672. doi:10.2337/diabetes.52.7.1664. PubMed: 12829630. 12829630

[14] Hoggard N, Mercer JG, Rayner DV, Moar K, Trayhurn P et al. (1997) Localization of leptin receptor mRNA splice variants in murine peripheral tissues by RT-PCR and in situ hybridization. Biochem Biophys Res Commun 232: 383–387. doi:10.1006/bbrc.1997.6245. PubMed: 9125186. 9125186

[15] Fei H, Okano HJ, Li C, Lee G-H, Zhao C et al. (1997) Anatomic localization of alternatively spliced leptin receptors (Ob-R) in mouse brain and other tissues. Proc Natl Acad Sci U S A 94: 7001–7005. doi:10.1073/pnas.94.13.7001. PubMed: 9192681. 9192681 PMC21274

[16] De Matteis R, Dashtipour K, Ognibene A, Cinti S (1998) Localization of leptin receptor splice variants in mouse peripheral tissues by immunohistochemistry. Proc Nutr Soc 57: 441-448. doi:10.1079/PNS19980063. PubMed: 9794002. 9794002

[17] Barrenetxe J, Villaro AC, Guembe L, Pascual I, Muñoz-Navas M, Barber A, Lostao MP (2002) Distribution of the long leptin receptor isoform in brush border, basolateral membrane, and cytoplasm of enterocytes. Gut 50: 797–802. doi:10.1136/gut.50.6.797. PubMed: 12010881. 12010881 PMC1773228

[18] Buyse M, Sitaraman SV, Liu X, Bado A, Merlin D (2002) Luminal leptin enhances CD147/MCT-1-mediated uptake of butyrate in the human intestinal cell line Caco_2_-BBE. J Biol Chem 277: 28182–28190. doi:10.1074/jbc.M203281200. PubMed: 12034734. 12034734

[19] Hansen GH, Niels-Christiansen LL, Danielsen EM (2008) Leptin and the Obesity Receptor (OB-R) in the Small Intestine and Colon: A Colocalization Study. J Histochem Cytochem 56: 677–685. doi:10.1369/jhc.2008.950782. PubMed: 18413648. 18413648 PMC2430163

[20] Sakar Y, Nazaret C, Lettéron P, Ait Omar A, Avenati M et al. (2009) Positive regulatory control loop between gut leptin and intestinal GLUT2/GLUT5 transporters links to hepatic metabolic functions in rodents. PLOS ONE 4: e7935. doi:10.1371/journal.pone.0007935. PubMed: 19956534. 19956534 PMC2780353

[21] Buyse M, Berlioz F, Guilmeau S, Tsocas A, Voisin T et al. (2001) PepT1-mediated epithelial transport of dipeptides and cephalexin is enhanced by luminal leptin in the small intestine. J Clin Invest 108: 1483–1494. doi:10.1172/JCI200113219. PubMed: 11714740. 11714740 PMC209419

[22] Lostao MP, Urdaneta E, Martínez-Ansó E, Barber A, Martínez JA (1998) Presence of leptin receptors in rat small intestine and leptin effect on sugar absorption. FEBS Lett 423: 302–306. doi:10.1016/S0014-5793(98)00110-0. PubMed: 9515728. 9515728

[23] Barrenetxe J, Barber A, Lostao MP (2001) Leptin effect on galactose absorption in mice jejunum. J Physiol Biochem 57: 345–346. doi:10.1007/BF03179829. PubMed: 12005038. 12005038

[24] Ducroc R, Sakar Y, Fanjul C, Barber A, Bado A, Lostao MP (2010) Luminal leptin inhibits L-glutamine transport in rat small intestine: involvement of ASCT2 and B0AT1. Am J Physiol Gastrointest Liver Physiol 299: G179–G185. doi:10.1152/ajpgi.00048.2010. PubMed: 20448142. 20448142 PMC3112213

[25] Fanjul C, Barrenetxe J, Iñigo C, Sakar Y, Ducroc R et al. (2012) Leptin regulates sugar and amino acids transport in the human intestinal cell line Caco-2. Acta Physiol 205: 82–91. doi:10.1111/j.1748-1716.2012.02412.x. PubMed: 22252010. 22252010

[26] Ducroc R, Guilmeau S, Akasbi K, Devaud H, Buyse M et al. (2005) Luminal leptin induces rapid inhibition of active intestinal absorption of glucose mediated by sodium-glucose cotransporter 1. Diabetes 54: 348–354. doi:10.2337/diabetes.54.2.348. PubMed: 15677491. 15677491

[27] In ˜igo C, Patel N, Kellett GL, Barber A, Lostao MP (2007) Luminal leptin inhibits intestinal sugar absorption in vivo. Acta Physiol 190: 303–310. doi:10.1111/j.1748-1716.2007.01707.x. 17488247

[28] Morton NM, Emilsson V, Liu YL, Cawthorne MA (1998) Leptin action in intestinal cells. J Biol Chem 273: 26194–26201. doi:10.1074/jbc.273.40.26194. PubMed: 9748302. 9748302

[29] Cammisotto PG, Bendayan M, San´e A, Dominguez M, Garofalo C et al. (2010). Receptor-Mediated Transcytosis of Leptin through Human Intestinal Cells In Vitro. Int J Cell Biol 2010: 2010:928169 10.1155/2010/928169 PMC2862316 20454702

[30] Stan S, Levy E, Bendayan M, Zoltowska M, Lambert M et al. (2001) Effect of human recombinant leptin on lipid handling by fully differentiated Caco-2 cells. FEBS Lett 508: 80–84. doi:10.1016/S0014-5793(01)03032-0. PubMed: 11707272. 11707272

[31] Hubatsch I, Ragnarsson EGE, Artursson P (2007) Determination of drug permeability and prediction of drug absorption in Caco-2 monolayers. Nat Protoc 2: 2111–2119. doi:10.1038/nprot.2007.303. PubMed: 17853866. 17853866

[32] Gould GW, Cuenda A, Thomson FJ, Cohen C (1995) The activation of distinct mitogen-activated protein kinase cascades required for the stimulation of 2-deoxyglucose uptake by interleukin-1 and insulin-like growth factor in KB cells. Biochem J 311: 735–738. PubMed: 7487926. 7487926 10.1042/bj3110735 PMC1136064

[33] Barros LF, Young M, Saklatvala J, Baldwin SA (1997) Evidence of two mechanisms for the activation of the glucose transporter GLUT1 by anisomycin. J Physiol 504: 517–525. doi:10.1111/j.1469-7793.1997.517bd.x. PubMed: 9401960. 9401960 PMC1159956

[34] Helliwell PA, Richardson M, Affleck J, Kellett GL (2000a) Stimulation of fructose transport across the intestinal brush-border membrane by PMA is mediated by GLUT2 and dynamically regulated by protein kinase C. Biochem J 350: 149-154. doi:10.1042/0264-6021:3500149. PubMed: 10926838. 10926838 PMC1221236

[35] Helliwell PA, Richardson M, Affleck J, Kellett GL (2000b) Regulation of GLUT5, GLUT2 and intestinal brush-border fructose absorption by the extracellular signal-regulated kinase, p38 mitogen-activated kinase and phosphatidylinositol 3-kinase intracellular signalling pathways: implications for adaptation to diabetes. Biochem J 350: 163-169. doi:10.1042/0264-6021:3500163. PubMed: 10926840. 10926840 PMC1221238

[36] Kellett GL, Helliwell PA (2000) The diffusive component of intestinal glucose absorption is mediated by the glucoseinduced recruitment of GLUT2 to the brush-border membrane. Biochem J 350: 155–162. doi:10.1042/0264-6021:3500155. PubMed: 10926839. 10926839 PMC1221237

[37] Van den Brink GR, O’Toole T, Hardwick JC, Van den Boogaardt DE, Versteeg HH et al. (2000) Leptin signaling in human peripheral blood mononuclear cells, activation of p38 and p42/44 mitogen-activated protein (MAP) kinase and p70 S6 kinase. Mol Cell Biol Res Commun 4: 144–150. doi:10.1006/mcbr.2001.0270. PubMed: 11281728. 11281728

[38] Cohen B, Novick D, Rubinstein M (1996) Modulation of insulin activities by leptin. Science 274: 1185–1188. doi:10.1126/science.274.5290.1185. PubMed: 8895466. 8895466

[39] Zhao AZ, Bornfeldt KE, Beavo JA (1998) Leptin inhibits insulin secretion by activation of phosphodiesterase 3B. J Clin Invest 102: 869–873. doi:10.1172/JCI3920. PubMed: 9727054. 9727054 PMC508951

[40] Sweeney G, Niu W, Kanani R, Klip A (2000) Regulation of the Na^+^/K^+^-pump by leptin in 3T3-L1 fibroblasts. Endocrinology 141: 1277–1280. doi:10.1210/en.141.3.1277. PubMed: 10698206. 10698206

[41] O’Rourke L, Yeaman SJ, Shepherd PR (2001) Insulin and leptin acutely regulate cholesterol ester metabolism in macrophages by novel signaling pathways. Diabetes 50: 955–961. doi:10.2337/diabetes.50.5.955. PubMed: 11334438. 11334438

[42] Berti L, Kellerer M, Capp E, Häring HU (1997) Leptin stimulates glucose transport and glycogen synthesis in C2C12 myotubes: evidence for a PI3-kinase mediated effect. Diabetologia 40: 606–609. doi:10.1007/s001250050722. PubMed: 9165231. 9165231

[43] Harvey J, McKay NG, Walker KS, Van der Kaay J, Downes CP, Ashford MLJ (2000) Essential role of phosphoinositide 3- kinase in leptin-induced KATP channel activation in the rat CRI-G1 insulinoma cell line. J Biol Chem 275: 4660–4669. doi:10.1074/jbc.275.7.4660. PubMed: 10671495. 10671495

[44] Hegyi K, Fu ¨lo¨ p K, Kovács K, Tóth S, Falus A (2004) Leptin induced signal transduction pathways. Cell Biol Int 28: 159–169. doi:10.1016/j.cellbi.2003.12.003. PubMed: 14984741. 14984741

[45] Banks AS, Davis SM, Bates SH, Myers MG Jr (2000) Activation of downstream signals by the long form of the leptin receptor. J Biol Chem 275: 14563–14572. doi:10.1074/jbc.275.19.14563. PubMed: 10799542. 10799542

[46] Attoub S, Noe V, Pirola L, Bruyneel E, Chastre E et al. (2000) Leptin promotes invasiveness of kidney and colonic epithelial cells via phosphoinositide 3-kinase-, Rho-, and Rac-dependent signaling pathways. FASEB J 14: 2329–2338. doi:10.1096/fj.00-0162. PubMed: 11053255. 11053255

[47] Sweeney G (2002) Leptin signaling. Cell Signal 14: 655–663. doi:10.1016/S0898-6568(02)00006-2. PubMed: 12020765. 12020765

[48] Berti L, Gammeltoft S (1999) Leptin stimulates glucose uptake in C2C12 muscle cells by activation of ERK2. Mol Cell Endocrinol 157: 121–130. doi:10.1016/S0303-7207(99)00154-9. PubMed: 10619403. 10619403

